# Low miR-143/miR-145 Cluster Levels Induce Activin A Overexpression in Oral Squamous Cell Carcinomas, Which Contributes to Poor Prognosis

**DOI:** 10.1371/journal.pone.0136599

**Published:** 2015-08-28

**Authors:** Andreia Bufalino, Nilva K. Cervigne, Carine Ervolino de Oliveira, Felipe Paiva Fonseca, Priscila Campioni Rodrigues, Carolina Carneiro Soares Macedo, Lays Martin Sobral, Marcia Costa Miguel, Marcio Ajudarte Lopes, Adriana Franco Paes Leme, Daniel W. Lambert, Tuula A. Salo, Luiz Paulo Kowalski, Edgard Graner, Ricardo D. Coletta

**Affiliations:** 1 Department of Oral Diagnosis, School of Dentistry, University of Campinas, Piracicaba-SP, Brazil; 2 Department of Dentistry, Federal University of Rio Grande do Norte, Natal-RN, Brazil; 3 Brazilian Biociences National Laboratory-CNPEM, Campinas-SP, Brazil; 4 Integrated Biosciences, School of Clinical Dentistry and Sheffield Cancer Centre, University of Sheffield, Sheffield, United Kingdom; 5 Department of Diagnostics and Oral Medicine, Institute of Dentistry and Oulu University Hospital and Medical Research Center, University of Oulu, Oulu and Institute of Dentistry, University of Helsinki, Helsinki, Finland; 6 Department of Head and Neck Surgery and Otorhinolaryngology, A. C. Camargo Cancer Center, São Paulo-SP, Brazil; The University of Hong Kong, CHINA

## Abstract

Deregulated expression of activin A is reported in several tumors, but its biological functions in oral squamous cell carcinoma (OSCC) are unknown. Here, we investigate whether activin A can play a causal role in OSCCs. Activin A expression was assessed by qPCR and immunohistochemistry in OSCC tissues. Low activin A-expressing cells were treated with recombinant activin A and assessed for apoptosis, proliferation, adhesion, migration, invasion and epithelial-mesenchymal transition (EMT). Those phenotypes were also evaluated in high activin A-expressing cells treated with follistatin (an activin A antagonist) or stably expressing shRNA targeting activin A. Transfections of microRNA mimics were performed to determine whether the overexpression of activin A is regulated by miR-143/miR-145 cluster. Activin A was overexpressed in OSCCs in comparison with normal oral mucosa, and high activin A levels were significantly associated with lymph node metastasis, tumor differentiation and poor survival. High activin A levels promoted multiple properties associated with malignant transformation, including decreased apoptosis and increased proliferation, migration, invasion and EMT. Both miR-143 and miR-145 were markedly downregulated in OSCC cell lines and in clinical specimens, and inversely correlated to activin A levels. Forced expression of miR-143 and miR-145 in OSCC cells significantly decreased the expression of activin A. Overexpression of activin A in OSCCs, which is controlled by downregulation of miR-143/miR-145 cluster, regulates apoptosis, proliferation and invasiveness, and it is clinically correlated with lymph node metastasis and poor survival.

## Introduction

Oral cavity cancers represent 6% of all diagnosed cancers worldwide, and oral squamous cell carcinoma (OSCC) is the most frequent, accounting for 90% of all cases at this site [1]. Despite continued improvements in the therapeutic strategies, mortality rates of OSCC continue to be high, giving rise to an overall 5-year survival rate of approximately 50% [1]. This low survival rate is due to an association of factors, including diagnosis at advanced-disease stage, high recurrence rates and our incomplete understanding of the molecular mechanisms responsible for oral tumorigenesis. Thus, elucidating the cellular and molecular mechanisms behind OSCC is mandatory for a better understanding of the genetic events associated with OSCC progression and to develop novel and individualized therapeutic approaches to this disease, which should ultimately provide an important impact on patient survival.

Activin A, the homodimeric protein encoded by the *INHBA* gene, is a multifunctional member of the transforming growth factor β (TGF-β) family with important roles in cell growth, differentiation and apoptosis in events related to angiogenesis, inflammation, immunity and embryogenesis [2]. As a result, defects in its expression have been linked to uncontrolled proliferation and survival, leading to cancer development and progression. Although deregulated expression of activin A has been broadly reported in a variety of cancers [3–5], its role in OSCCs is not yet well understood. In a recent study our group demonstrated that immunodetection of activin A correlates with occult lymph node metastasis in patients with early OSCCs of the tongue and that its expression is an independent marker of patient outcome, supporting a role of activin A as a prognostic marker of OSCCs [6]. Additionally, we showed that carcinoma-associated fibroblasts (CAFs) promote tumorigenesis of OSCC cell lines via secretion of activin A [7]. Furthermore, overexpression of activin A in OSCCs was associated with increased regional lymph node metastasis and lower patient survival [8].

In this study we confirm the prognostic significance of activin A overexpression in OSCCs and examine the molecular mechanism by which activin A influences oral tumorigenesis. We show that activin A overexpression in OSCCs is significantly correlated with regional lymph node metastasis and poorly differentiated tumors, and patients with high expression of activin A show shortened survival. In vitro analysis revealed that activin A blocks apoptosis whereas it controls proliferation via regulation of p16, p21 and p27. Our data also demonstrate that activin A promotes motility and invasiveness of OSCC cells, as well as epithelial-mesenchymal transition (EMT), as revealed by modulation of the expression of EMT markers E-cadherin, N-cadherin and vimentin. Finally, we showed that expression of the miR-143/miR-145 cluster is inversely correlated with INHBA levels in OSCC cell lines and specimens, and overexpression of those microRNAs downregulated INHBA mRNA.

## Materials and Methods

### INHBA mRNA levels in previously published microarrays

To examine the expression pattern of INHBA in published microarray data, we performed a metanalysis using data mining from the Oncomine Research Premium Edition database (https://www.oncomine.org). The first step was to identify previously published microarray gene expression data comparing normal oral mucosa and OSCC. Filters for selection of the data were studies that included INHBA in the analysis, comparing cancer vs normal tissue, cancer type (squamous cell carcinoma) and primary tumor sites in the oral cavity. After applying those filters, we ended up with 9 datasets (312 samples) from published studies. The expression level was considered the median rank for the gene across each of the analysis, and the given p-value was based in the median-ranked analysis at a cut off 0.01 (p<0.01).

### Samples and clinicopathological data

To confirm the overexpression of activin A in OSCCs, fresh samples of OSCC (n = 17) and normal oral mucosa (n = 11) were used to investigate the expression of INHBA using quantitative PCR (qPCR). Those samples were also used for the expression of the putative microRNA regulators of INHBA mRNA (see below). The samples were snap frozen in liquid nitrogen and kept at -80°C until use. The initial diagnosis was based on clinical findings and confirmed later by histopathological analysis of the specimens.

Using immunohistochemistry analysis, we also investigated the association of activin A expression with clinicopathological features of 115 OSCCs, which were obtained from patients treated at the Department of Head and Neck Surgery and Otorhinolaryngology, A.C. Camargo Cancer Center, São Paulo, Brazil. The OSCC patients (92 males and 23 females) showed a mean of 55.6 ± 10.4 years (ranged 31–79 years). History of alcohol consumption was recorded in 91 patients (79.1%) and tobacco smoking in 106 (92.2%) patients. The site of the primary tumor was predominantly the tongue (n = 82) and other sites such as the floor of mouth (n = 10), gingiva (n = 10), buccal mucosa (n = 8) and the retromolar region (n = 5) accounted for the remaining cases. The patients were staged according to the International Union Against Cancer (TNM stage) as follows: T1 (n = 10), T2 (n = 31), T3 (n = 31) and T4 (n = 43), as well as N0 (n = 50) and N+ (n = 65). All patients were staged as M0 at the time of diagnosis. Regarding treatment, surgery as monotherapy was performed in 35 patients, 72 were treated by combination of surgery and postoperative radiotherapy, and 8 patients were treated by surgery and postoperative radiotherapy and chemotherapy. Tumors were histopathologically classified according to the World Health Organization (WHO) grading system, and demonstrated a distribution of 17 well differentiated, 42 moderately differentiated and 56 poorly differentiated tumors. Surgical margin, identified as the closest distance between the tumor and the surgical resection edge (both in deep muscle and lateral on mucosa), was categorized into 2 groups based on cut-off value of 5 mm. Margins of less than 5 mm were considered involved (n = 14), and margins of 5 mm or more were classified as free (n = 101). Vascular and perineural invasions were categorized as present or absent. After treatment, patients were followed up for at least 5 years and disease recurrence was histologically confirmed. The outcomes were categorized as overall survival, time from treatment initiation until death or last follow-up information, and disease-free survival, time from treatment initiation until diagnosis of the first recurrence (local, regional or distant) or last follow up information for those without recurrence. Patients had signed an informed consent form prior to participation in this study, which was approved by the Human Research Ethics Committee of the School of Dentistry, University of Campinas, Brazil (protocol number 031/2011).

### Cell culture

HaCat, an immortalized but not transformed epithelial cell line, was maintained in DMEM (Invitrogen, USA) containing 10% fetal bovine serum (FBS) and antibiotics at 37°C in a 5% CO_2_ air atmosphere. Normal human gingival keratinocyte (HGK) cell line was cultured in serum-free, low calcium medium (Gibco’s Keratinocyte-SFM; Invitrogen, USA) containing specific supplements and antibiotics in a humidified atmosphere of 5% CO_2_. The human OSCC cell lines SCC-4, SCC-9, SCC-15 and SCC-25 were obtained from American Type Culture Collection (ATCC, Manassas, VA, USA), and cultured as recommended in a 1:1 mixture of Dulbecco’s modified Eagle’s medium and Ham’s F12 medium (DMEM/F12; Invitrogen, USA) supplemented with 10% fetal bovine serum (FBS), 400 ng/ml hydrocortisone (Sigma-Aldrich, USA) and antibiotics. The SCC-9 ZsGreen LN-1 cell line, isolated from a metastatic cervical lymph node, was previously described [9] and cultured in the same conditions as the parental cell line. HSC-3, a human squamous cell carcinoma cell line of the tongue (JCRB 0623; Osaka National Institute of Health Sciences, Japan), was cultured in DMEM/F-12 medium (Invitrogen, USA) supplemented with 10% FBS, 50 μg/ml ascorbic acid (Sigma-Aldrich, USA), 400 ng/ml hydrocortisone (Sigma-Aldrich, USA) and antibiotics.

### qPCR

Total RNA from fresh tissues and cell lines was isolated with the RNeasy mini kit (Qiagen, USA) or the mirVana miRNA isolation kit (Ambion, USA), according to the manufacturer's protocols. Following DNase I treatment in order to eliminate genomic DNA contamination, 1 μg of total RNA per sample was used to generate cDNA using Oligo-dT (Invitrogen, USA) and reverse transcriptase (Superscript II RT enzyme, Invitrogen, USA). The resulting cDNAs were subjected to qPCR using specific primers and SYBR Green PCR master mix (Applied Biosystems, USA) in the StepOnePlus Real Time PCR (Applied Biosystems, USA). Gene expression was determined using the delta-delta CT method and the housekeeping gene PPIA (cyclophilin A) was used as reference gene for data normalization. All reactions were performed in triplicate. Pairs of primers are described in S1 Table.

### Immunohistochemistry

Activin A immunostaining was performed using the streptavidin-biotin peroxidase complex method. Briefly, after dewaxing and hydration in graded alcohol solutions, the sections were treated with 3% H_2_O_2_ followed by antigen retrieval with 10 mM citric acid pH 6.0 in a pressure cooker. After washing with phosphate-buffered saline (PBS), the sections were treated with 1% bovine serum albumin (BSA) in PBS for 1 h and then incubated with polyclonal rabbit antibody against activin A (R&D Systems, USA), diluted 1:100, followed by the LSAB method (LSAB+ System-HRP kit, Dako, USA). Reactions were developed by incubating the sections with 0.6 mg/ml 3,3'-diaminobenzidine tetrahydrochloride (Sigma-Aldrich, USA) containing 0.01% H_2_O_2_. Control reactions were performed by omission of the primary antibody.

Activin A expression was assessed with the aid of the Aperio ScanScope CS (Aperio Technologies, USA). Briefly, glass slides were scanned into high-resolution images, which were analyzed in the Pixel Count V9 algorithm software (Aperio Technologies, USA). The tumor cell islands were delimitated and by using specific input parameters (hue value = 0.01, hue width = 0.49, color saturation threshold = 0.025 and intensity threshold ranging from 100 to 205), the percentage of cytoplasm positivity was calculated and classified in three range categories, according to its staining intensity as weak (from 175 to 205), moderate (from 101 to 174) and strong (from 0 to 100). To each category, an intensity score was set: 1 for weak, 2 for moderate, and 3 for strong staining. Tumor final scores were calculated as the sum of the percentage of each category multiplied by its intensity score, using the following equation: [(%weak x 1) + (%moderate x 2) + (%strong x 3)].

### Treatments

Lyophilized recombinant activin A and follistatin (R&D Systems, USA) were dissolved in culture medium, aliquoted and stored at -80°C. To assess the effect of activin A, cells were cultured in 0.1% FBS media containing 0, 1, 10 or 100 ng/ml for 24 h. Follistatin was used at concentration of 100 ng/ml.

### Stable cells mediating INHBA silence

SCC-9 ZsGreen LN-1 cells grown in a 12-well plate at confluence of 50% were incubated with control or INHBA shRNA lentiviral particles (MISSION shRNA Lentiviral Transduction Particles, Sigma-Aldrich, USA) in culture media containing 8 μg/ml of polybrene (Sigma-Aldrich, USA) for 8 h. After washing with PBS, cells were cultured in fresh media for an additional period of 48 h. Cells were then split in a 1:5 concentration, and cultured for 10 days in the presence of 1 μg/ml of puromycin dihydrochloride (Sigma-Aldrich, USA) to select resistant cells. The efficacy of INHBA knockdown was determined by qPCR and enzyme-linked immunosorbent assay (ELISA).

### ELISA

Conditioned cell culture media was collected and the cells harvested using 0.25% trypsin and counted with a cell counter (Countess Automated Cell Counter, Invitrogen, USA). After centrifugation, microtiter plate wells were coated with 100 μl of the conditioned-media for 2 h at room temperature. The wells were then washed 3 times with 400 μl of 1% Tween 20 in PBS and nonspecific binding sites were blocked with 3% BSA in PBS for 2 h. After washing, monoclonal mouse antibody anti-activin A (clone 69403, R&D Systems, USA), diluted 1:100 in PBS, was added to the wells and incubated for 2 h. After another washing step, peroxidase-conjugated anti-mouse IgG (Vector Labs, USA) diluted 1:1000 in PBS was added and maintained for 1 h. The reaction was developed with 0.5 mg/ml of o-phenylenediamine (Sigma-Aldrich, USA) in 0.5 M citric buffer pH 5.5 containing 0.01% H_2_O_2_ for 20 min. After terminating the reaction with 50 μl of 2 N H_2_SO_4_, absorbance was read at 450 nm with λ correction at 570 nm. Activin A levels, as represented by absorbance values, were calculated by dividing these values by the number of cells/well.

### Apoptosis analysis

The apoptosis index was determined by annexin V labeling. Briefly, cells were harvested, washed and resuspended in the binding buffer (10 mM HEPES pH 7.4, 150 mM NaCl, 5 mM KCl, 1 mM MgCl_2_ and 1.8 mM CaCl_2_) containing annexin V-PE and 7-AAD (BD Biosciences, USA). Apoptosis was analyzed on a FACScalibur flow cytometer equipped with an argon laser (BD Biosciences, USA) and quantified as the number of annexin V-PE positive and 7-AAD negative cells divided by the total number of cells. A minimum of 10,000 events was analyzed for each sample.

To confirm the inhibitory effects of activin A, in a second set of experiments, apoptosis was induced with 0.03 μM staurosporine (Invitrogen, USA) during the last 4 h of activin A treatment, following annexin V labeling.

### Bromodeoxyuridine-labeling (BrdU) index

Cells were plated in 96-well plates at a density of 10,000 cells per well in 100 μl of media containing 10% FBS. After 16 h, the cells were washed with PBS and cultured in serum-free media for an additional 24 h. Following serum starvation, the media were replaced by media containing 10% FBS. Proliferation rates were determined 24 h after incubation by measuring BrdU incorporation into DNA using the cell proliferation ELISA BrdU (colorimetric) kit (Roche Applied Science, USA).

### Cell cycle analysis

Cells were synchronized for 24 h by serum starvation and released with media containing 10% FBS. After 24 h, cells were collected, fixed in 70% ethanol for 30 min, treated with 10 μg/ml of RNAse (Sigma-Aldrich, USA) and stained with 50 μg/ml of propidium iodide (Sigma-Aldrich, USA). The distribution of cells in the cell cycle phases was analyzed with the aid of the FACSCalibur flow cytometer (BD Biosciences, USA) equipped with an argon laser and the ModFit LT software (Verity Software House, USA).

### Western blot

Western blot analysis was used to determine the expression of proteins related to G1 phase of the cell cycle, and also to confirm the effects of activin A on EMT markers. Cells were washed with cold PBS and lysed in a buffer containing 20 mM Tris-HCl pH 8.0, 137 mM NaCl, 10% glycerol, 2 mM EDTA, and protease inhibitors. After centrifugation, protein concentrations were measured using a protein assay according to the manufacturer’s instructions (Bio-Rad Protein Assay, Bio-Rad, USA). Thirty μg of total proteins per sample were resolved in a 10% SDS-PAGE under reducing conditions, and transferred to nitrocellulose membranes. The membranes were blocked with 10% non-fat dry milk in PBS containing 0.1% Tween-20, rinsed in the same buffer, and incubated for 2 h with the primary antibodies (S2 Table). After washing, the protein bands were detected using enhanced chemiluminescence (ECL) Western Blotting System (GE Healthcare, USA).

### Adhesion assay

Wells of a 96-well culture plate were coated with 10 μg/cm^2^ of type I collagen, fibronectin or laminin (BD Biosciences, USA) in 100 μl of PBS for 24 h at 4°C. The wells were washed 3 times with 200 μl of PBS and then coated with the same volume of 3% BSA in PBS for 2 h at 37°C. Control wells were coated only with 3% BSA solution. Cells were harvested and then resuspended in DMEM supplemented with 3% BSA at a final concentration of 3,000 cells in 100 μl. The wells were washed and 100 μl of the cell suspension was added to each well. The plate was incubated for 1 h at 37°C in 5% CO_2_. Non-adherent cells were washed away and remaining adhered cells were fixed with 10% formalin for 15 min and stained with 1% toluidine blue in 1% borax solution. Absorbance was measured at 650 nm.

### Hanging drop assay

Hanging drop assay was performed to estimate the cell-cell adhesive properties of activin A-silenced cells, as described previously with few modifications [10]. Briefly, 2x10^4^ cells were suspended in 27 μl drops of complete media and kept in the lid of 35 mm plate for 16 h at 37°C in a 5% CO_2_ air atmosphere. Images of cell aggregates of 5 random fields from 5 different suspensions were visualized with a confocal microscopy in sequential Z-stack images (LEICA TCS SP5, Leica Microsystems, Germany).

### Migration and invasion assays

Transwell migration and invasion assays were performed in 6.5 mm inserts with 8 μm pore size (Corning, USA). For invasion assay, membranes were coated with 50 μl of growth factor-reduced matrigel (BD Biosciences, USA). Serum starved cells (80,000 cells/well) were plated into the upper chamber in 200 μl of serum-free DMEM. As chemoattractant, 500 μl of complete medium was used in the lower chamber. Experiment times varied between 24 h for migration assays and 72 h for invasion assays. Assessment of migration or invasion was performed by gently removing cell in the interior part of the insert with a cotton swab. Cells on the bottom of the membrane were fixed in 10% formalin for 15 min and stained with 1% toluidine blue in 1% borax solution. The excess dye was washed out and cells were then eluted in 1% SDS solution for 5 min. Absorbance was measured at 650 nm.

### Quantification of filopodia and lamellipodia

Cells grown in cell culture glass slides (Lab-Tek, Thermo Scientific, USA) were fixed in 4% paraformaldehyde in PBS for 10 mim and then permeabilized with 0.5% Triton X-100 in PBS for 10 min. Following, cells were incubated with Alexa Fluor 488 phalloidin (diluted 1:200, Invitrogen, USA) and DRAQ5 (diluted 1:1000, BioStatus Limited, United Kingdon) for 1 h. Quantification of filopodia and lamellipodia was performed with images captured with a confocal microscopy (LEICA TCS SP5, Leica Microsystems, Germany).

### miR-143 and miR-145 expression analysis

The expression of miR-143 and miR-145 was assessed in cell lines and fresh tumor specimens. Briefly, 1 μg of total RNA was converted into specific cDNA derived from mature microRNAs using TaqMan microRNA Reverse Transcription Kit (Applied Biosystems, USA) and quantified in triplicate using the TaqMan microRNA assay. The small nucleolar RNA (snoRNA) RNU48 was used as endogenous control. All assays were obtained from Applied Biosystems through their Assay-on-Demand service. Data were quantified and analyzed using sequence detection system (version 2.3) (Applied Biosystems, USA). The microRNA relative expression in fresh tumor specimens was normalized against endogenous control and pooled normal oral mucosa samples, and in the cell lines against endogenous control and HGK cells [11].

### Effect of miR-143 and miR-145 mimics on INHBA expression

SCC-9 and SCC-9 ZsGreen LN-1 cells were transfected with miR-143 or miR-145 mimics using the RNAiMAX reagent (Invitrogen, USA) as per the manufacturer's instructions. As control, cells were transfected with an unspecific scramble sequence (Pre-miR Negative Control #1, Life Technologies, USA). After 72 h, cells were harvested and subjected to qPCR and ELISA for quantification of INHBA as described above.

### Statistical analysis

Differences on expression of INHBA between fresh OSCC and normal oral mucosa samples were analyzed using the Mann-Whitney U test. Correlations between immunohistochemical expression of activin A and clinicopathological parameters of the tumors were performed using Spearman’s rank correlation. Survival curves were constructed based on the Kaplan-Meier method and compared with the Log-rank test. For multivariate survival analysis, the Cox proportional hazard model with a stepwise method including all parameters was employed.

All in vitro assays were performed at least three times in triplicates or quadruplicates. For those assays, Mann-Whitney U test or one-way analysis of variance (ANOVA) with post-hoc comparisons based on the Tukey's multiple comparisons test were applied. The level of significance considered was 5% (p≤0.05).

## Results

### INHBA is overexpressed in OSCCs compared to normal oral mucosa

**Fig 1:**
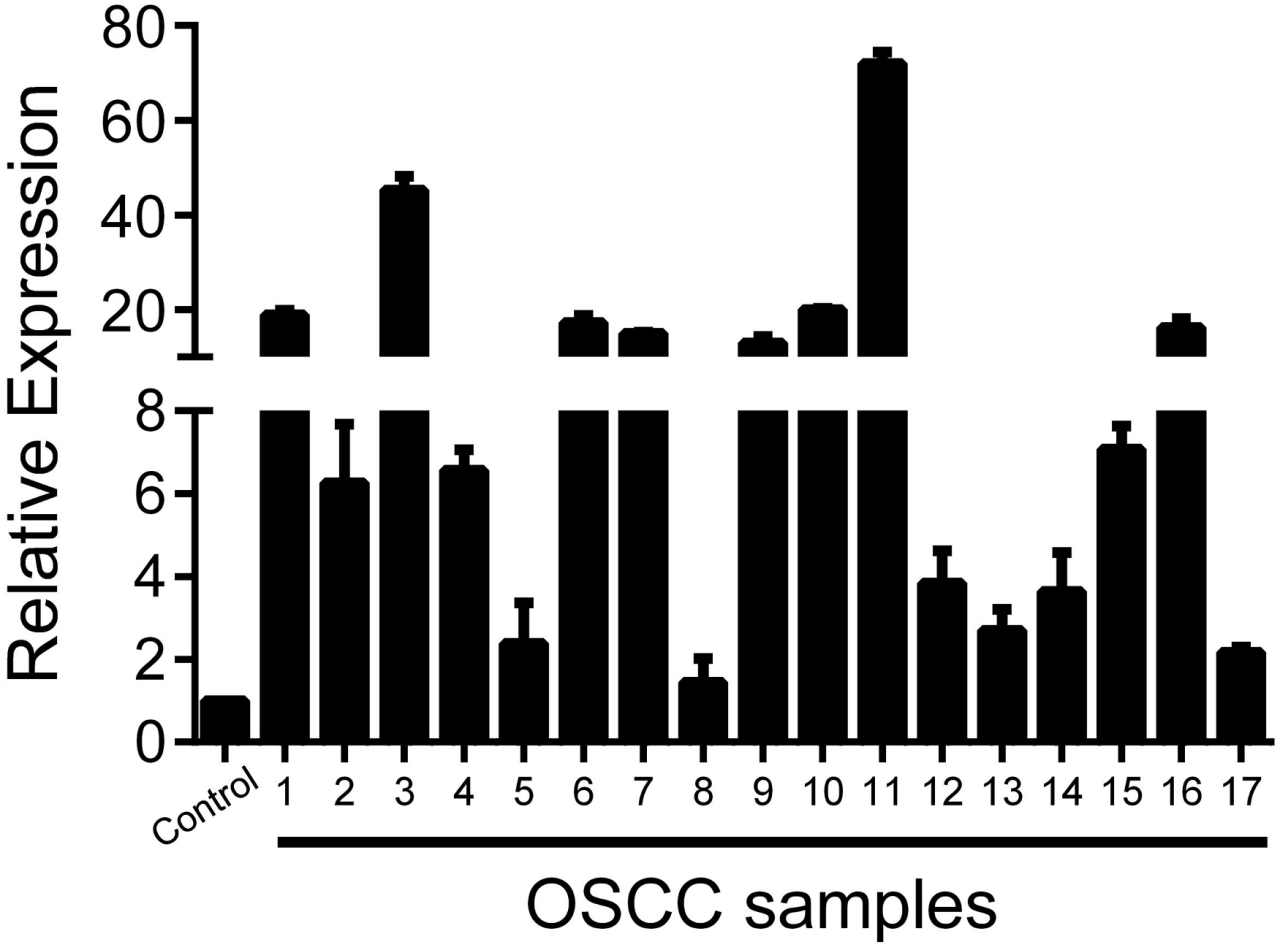
Activin A is overexpressed in OSCCs. The levels of INHBA mRNA in 17 fresh samples of OSCC and 11 normal oral mucosa samples were assessed by real-time PCR. Expression was normalized to the average value of the normal oral tissues. Data showed a clear overexpression above 2-fold in 16 out of 17 OSCC specimens (p = 0.0001).

To evaluate the expression of activin A in OSCCs, we first performed a metanalysis with published microarray data compiled from Oncomine Research Premium Edition database. Comparison across microarray gene expression studies showed that INHBA mRNA is significantly overexpressed in OSCC samples compared with normal oral mucosa (p = 0.0005). In line with this observation, we also detected a significantly higher content of INHBA mRNA in fresh OSCCs in comparison to normal control tissues (p = 0.0001; Fig 1). In this specific analysis, we used a pool of 11 normal oral tissues as a reference. Sixteen out of 17 OSCC samples showed, at least, 2-fold increased INHBA mRNA levels compared to the reference pool. Based on those results, we next analyzed the biological effects of activin A in OSCCs.

### Overexpression of activin A is significantly associated with shortened survival

**Fig 2:**
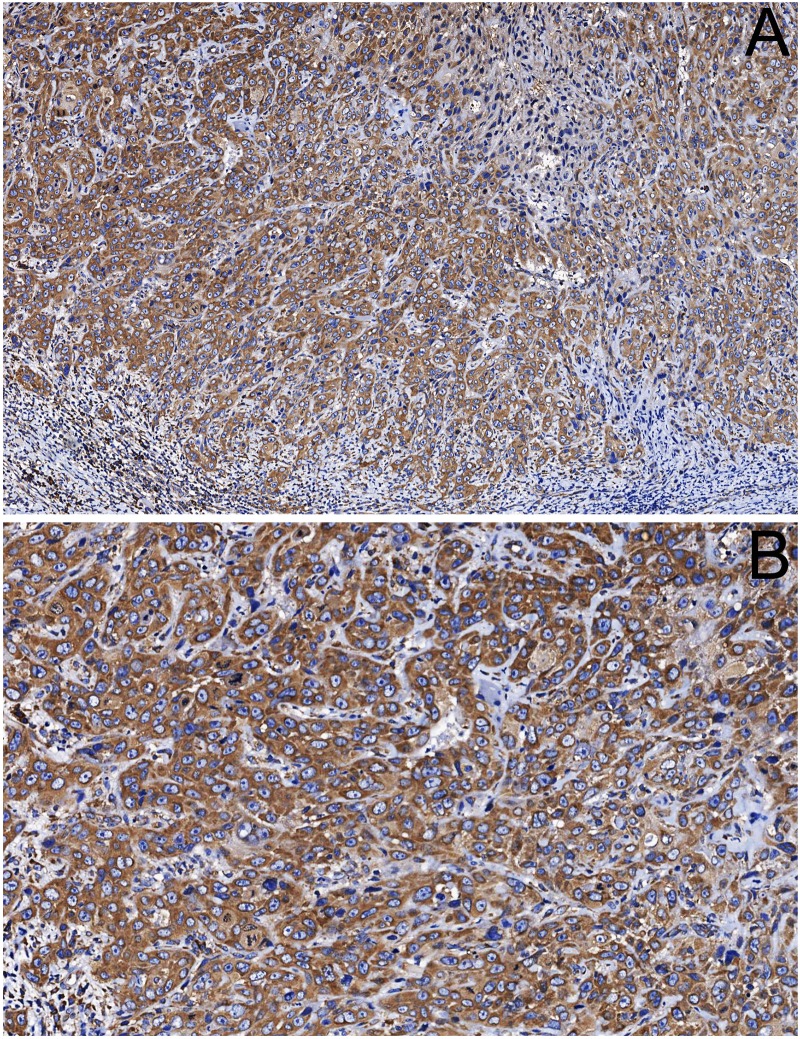
Activin A immunodetection in OSCC. (A) Positivity for activin A was observed in the cytoplasm of the tumor cells and in few stromal cells adjacent the tumor. (B) High power view revealed that tumor cells demonstrate variable expression of activin A even in the same tumor. (original magnification: A x100 and B x200).

To investigate whether activin A expression is associated with clinicopathological features of OSCC patients, we performed immunohistochemistry in 115 human OSCCs. Activin A was observed as a cytoplasmatic stain with variable distribution and intensity in the tumor cells (Fig 2). Immunoreactivity was also found in stromal cells, including inflammatory cells, CAFs and endothelial cells. The clinicopathological correlations with the expression of activin A in the tumor cells are described in Table 1. A significant correlation between activin A expression in the tumor cells and cervical lymph node metastasis (N stage) was observed, in that patients with metastasis (N+) had significantly higher activin A expression (p = 0.02). A significant correlation between high activin A expression and histopathological grade of the tumors was also found (p = 0.03). Approximately 60% of the tumors classified as poorly differentiated demonstrated high levels of activin A, while only 29% of the well differentiated tumors showed high activin A levels.

**Table 1:** Spearman correlation between activin A immunohistochemical expression and clinicopathological variables.

Variables	Correlation Coefficient	p value
Age	0.025	0.79
Gender	-0.013	0.89
Ethnic group	-0.017	0.85
Smoking habit	-0.052	0.58
Drinking habit	-0.067	0.47
Tumor site	0.075	0.42
T stage	0.082	0.38
N stage	0.216	0.02
Treatment	0.111	0.23
Histopathological grade	0.207	0.03
Surgical margin status	-0.001	0.98
Vascular invasion	-0.023	0.81
Perineural invasion	0.037	0.69
Recurrence	0.026	0.77

**Fig 3:**
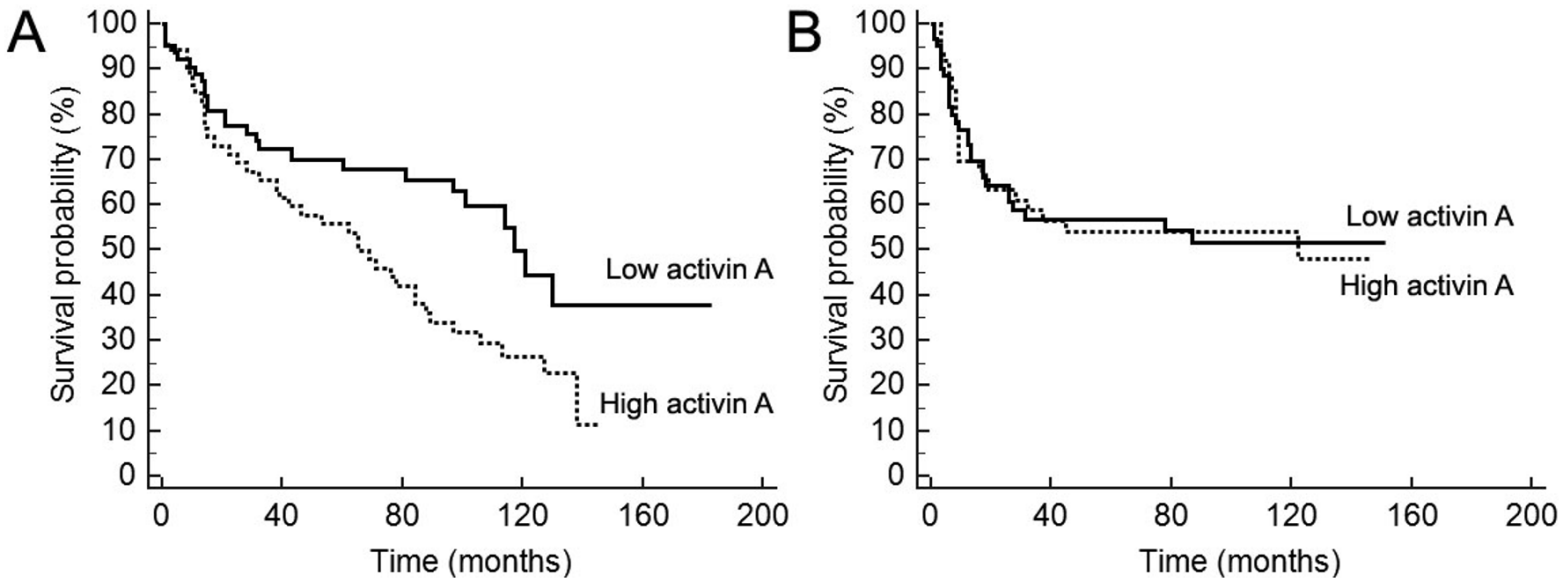
Survival curves based on immunoexpression of activin A. (A) Overall survival, as determined by the period between the treatment beginning until death or last follow-up information, and (B) disease-free survival, time between initiation of treatment until diagnosis of the recurrence (local, regional or distant) or last follow up information for those without recurrence. Censored deaths are indicated by vertical lines. The univariate analysis revealed that high positivity for activin A is significantly associated with shortened overall survival (p = 0.01).

High activin A immunoreactivity was a marker of reduced overall survival with a 5-year survival of 53.8% (95% CI 46.9–60.7) for the patients with strong positivity for activin A compared with 67.9% (95% CI 61.8–74.1) for those with low activin A expression (p = 0.01; Fig 3A). No significant influence of activin A immunoexpression in relapse (disease-free survival) was observed in this cohort (Fig 3B). To assess the independent predictive value of activin A, multivariate Cox-regression analysis of overall survival related to its expression levels in combination with other clinicopathological parameters was performed (Table 2). This analysis revealed that activin A and vascular infiltration were independent prognostic factors of this OSCC cohort. For activin A, a hazard ratio (HR) of 2.59 (95% CI 1.57–4.28, p = 0.0002) was found for high activin A immunopositivity in reference to low activin A levels. Similarly, presence of vascular infiltration revealed a HR of 1.94 (95% CI 1.18–3.21, p = 0.001).

**Table 2:** Cox multivariate analysis of factors associated with risk of death.

Parameter	Overall survival		
	HR	95% CI	p value
Activin A expression			
Low	Reference		
High	2.59	1.57–4.28	0.0002
Vascular invasion			
No	Reference		
Yes	1.94	1.18–3.21	0.001

### High levels of activin A suppresses apoptosis

To better understand the role of activin A in the events that control oral tumorigenesis, HaCat cells, which showed the lowest expression of activin A (S1 Fig), were treated with different concentrations of activin A (1, 10 and 100 ng/ml) for 24 h. In order to support the specific findings, two approaches were realized: treatment with 100 ng/ml of follistatin, an antagonist of activin A, and mRNA silencing using interference RNA. For both approaches, SCC-9 ZsGreen LN-1 cells, which showed the highest activin A expression levels (S1 Fig), were selected. SCC-9 ZsGreen LN-1 cells transducted with lentivirus carrying a specific sequence against INHBA demonstrated a significant reduction in both mRNA and protein levels in comparison with cells transducted with the control-sequence (S2 Fig).

**Fig 4:**
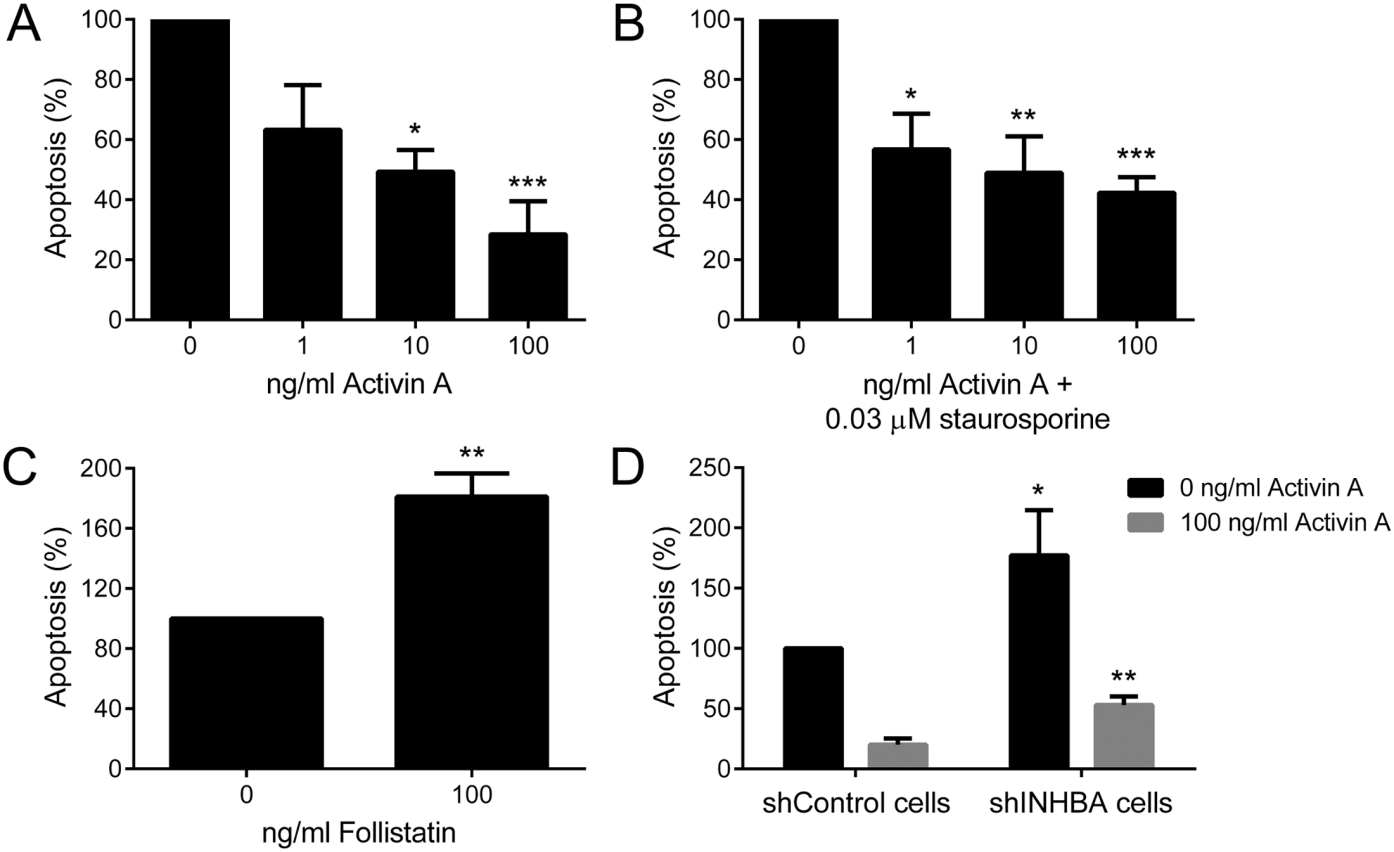
Activin A controls apoptosis of OSCC cells. Apoptotic cells were stained with annexin V-PE and propidium iodide and analyzed by flow cytometry. (A) Activin A blocked apoptosis of HaCat cells, reaching significant levels at concentration of 10 and 100 ng/ml. (B) Activin A reduced significantly apoptosis induced by staurosporine. Cells were treated with increased concentrations of activin A for 24 h, but in the last 4 h 0.03 μM of staurosporine were added to the cells. (C) Follistatin significantly induced apoptosis of SCC-9 ZsGreen LN-1 cells. (D) Activin A stable knockdown in SCC-9 ZsGreen LN-1 cells was significantly associated with increased levels of apoptosis. This phenotype was partially rescued adding 100 ng/ml of activin A. Bars represent the means ± SD of three independent experiments. *p>0.05, **p>0.01, ***p>0.005.

Compared with untreated cells, activin A blocked apoptosis in a concentration-dependent manner (Fig 4A). The decrease in the amount of apoptotic cells was first significant at concentration of 10 ng/ml of activin A and remained so at 100 ng/ml. To confirm this protective effect, HaCat cells under 24 h treatment with activin A were exposed to 0.03 μM of staurosporine, an inductor of apoptosis, in the last 4 h of treatment. Activin A was able to reduce significantly the number of apoptotic cells in a dose-dependent manner, reaching the maximum reduction (approximately 2/3) at concentration of 100 ng/ml (Fig 4B). Conversely, the number of apoptotic cells was significantly increased after 24 h treatment of SCC-9 ZsGreen LN-1 cells with follistatin (Fig 4C). Similarly, knock down of activin A promoted apoptosis in SCC-9 ZsGreen LN-1 cells, which was partially rescued with 100 ng/ml activin A (Fig 4D).

### Activin A promotes proliferation via regulation of cyclin-dependent kinase (CDK) inhibitors

**Fig 5:**
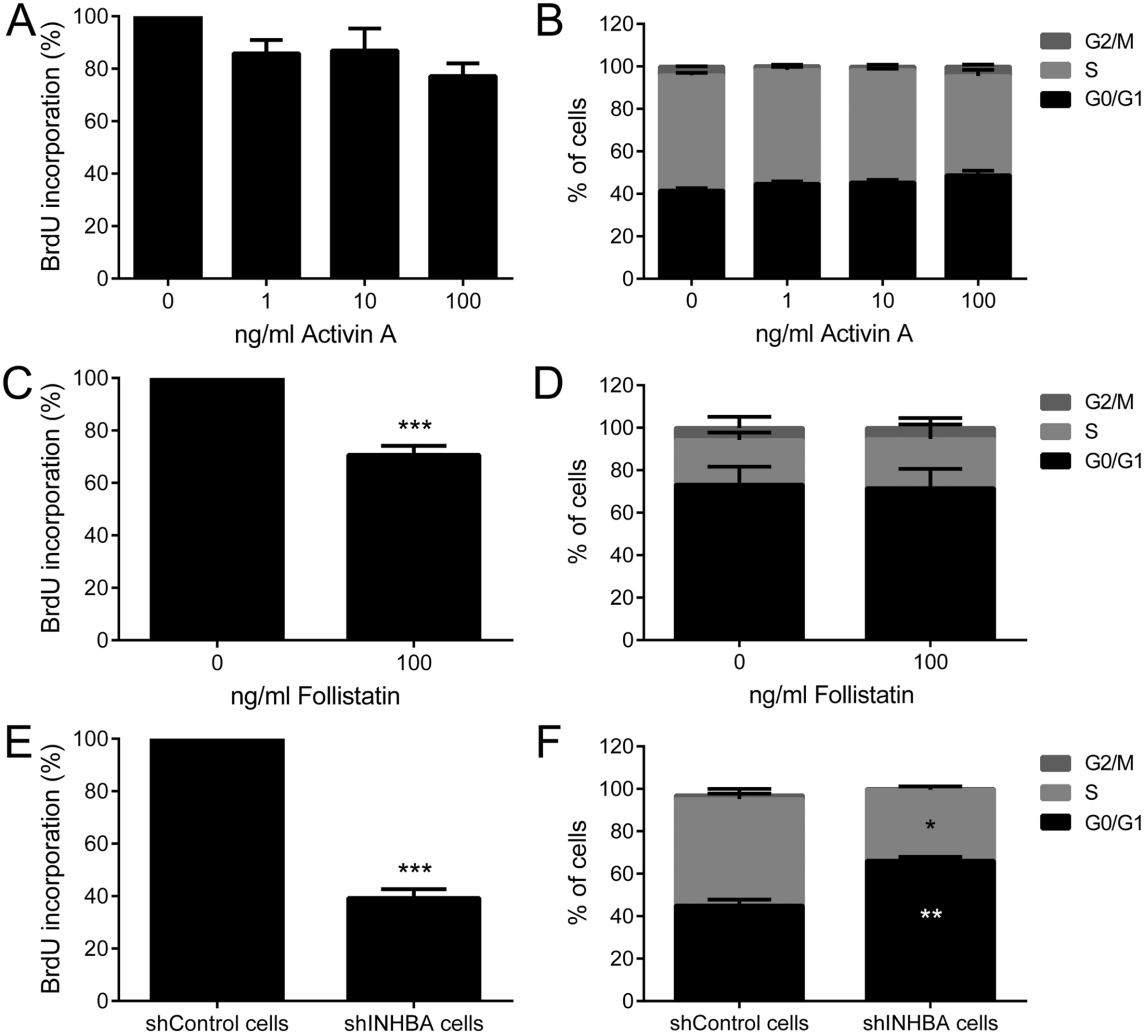
Downregulation of activin A leads to a decrease in proliferation. Treatment with recombinant activin A was not able to promote proliferation of HaCat cells, as revealed by bromodeoxyuridine (BrdU) incorporation index (A) and cell cycle analysis (B). (C) Follistatin at 100 ng/ml significantly blocked BrdU incorporation in SCC-9 ZsGreen LN-1 cells, but no effects on cell cycle distribution were observed (D). Knockdown of activin A significantly decreased proliferation (E), enhancing the number of cells at G0/G1 and reducing the number in the S phase of cell cycle (F). Bars represent the means ± SD of three independent experiments. *p<0.01, **p<0.001, ***p<0.0001.

Although no significant effects on proliferation, as evaluated by BrdU and cell cycle analysis, were observed after activin A treatment of HaCat cells (Fig 5A and 5B), follistatin reduced significantly the proliferation of SCC-9 ZsGreen LN-1 cells (Fig 5C and 5D). The difference between follistatin-treated and untreated cells was small, but statistically significant (p<0.01, Fig 5C). The specific shRNA against activin A drastically reduced the proliferation of SCC-9 ZsGreen LN-1 cells (p<0.001, Fig 5E). Accordingly, activin A downregulation promoted a significant increase of the number of cells at G0–G1 phases (p<0.001) and a clear reduction at S phase (p<0.01) in comparison with control (Fig 5F).

**Fig 6:**
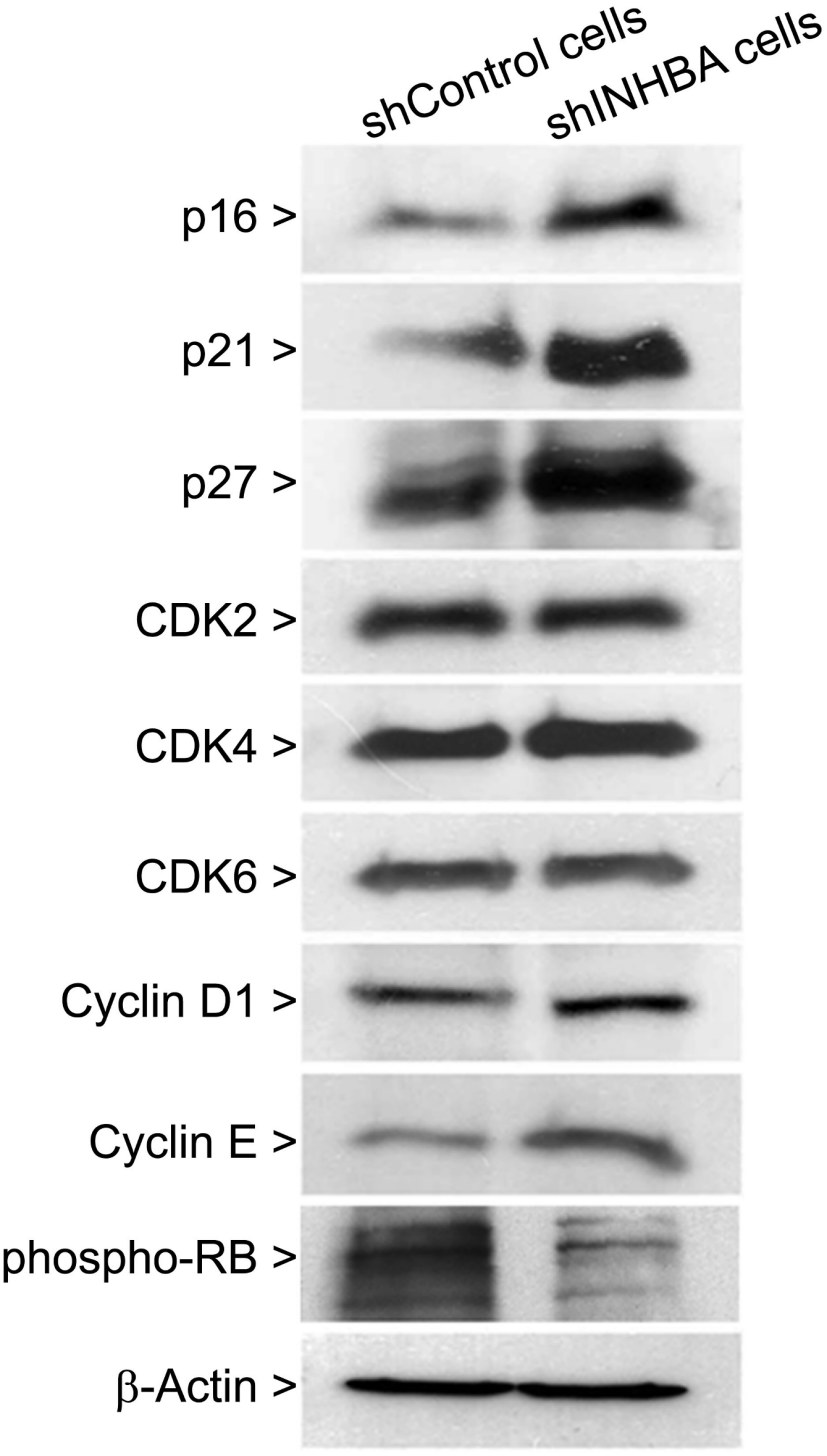
Knockdown of activin A increases cyclin-dependent kinase inhibitors p16, p21 and p27. Cells were harvested, lysed and proteins were subjected to western blot analysis using specific antibodies against p16, p21, p27, CDK2, CDK4, CDK6, cyclin D1, cyclin E and phospho-RB. β-actin was used as loading control. A strong increase in expression of p16, p21 and p27, concomitant with decrease in phosphorylation of RB, was observed in shINHBA cells in comparison with shControl cells. Beta-actin was used as loading control.

To further characterize the effects of activin A knock down on cell cycling, protein related to G1-S transition were analyzed using western blot. As shown in Fig 6, knock down of activin A was associated with increased expression of CDK inhibitors p16, p21 and p27 and a decreased phosphorylation of RB. The levels of CDK2, CDK4, CDK6, cyclin D1 and cyclin E were unaffected.

### Overexpression of activin A induces EMT and invasion

**Fig 7:**
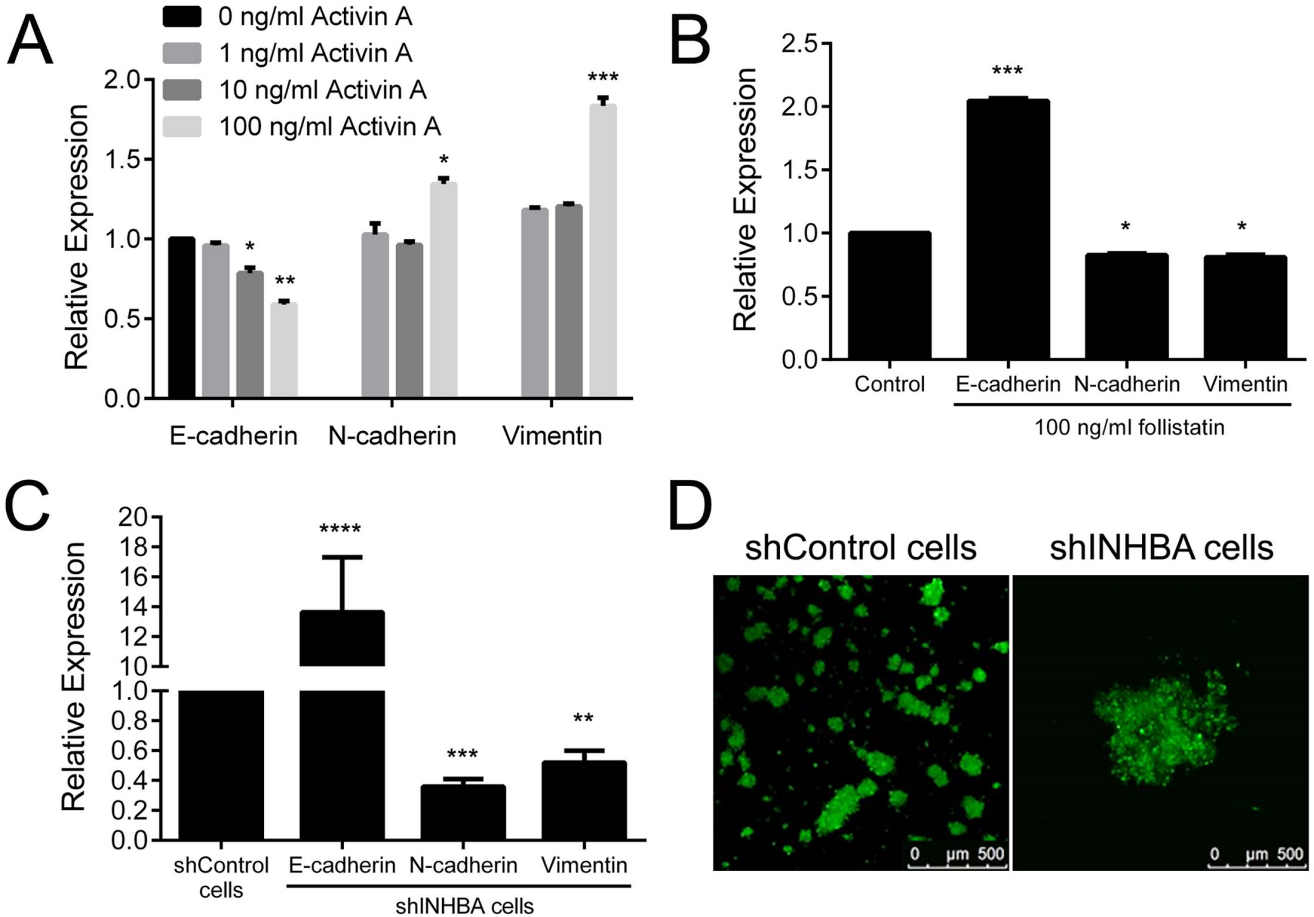
Activin A induces epithelial-mesenchymal transition (EMT). Levels of E-cadherin, N-cadherin and vimentin were analyzed by qPCR. (A) Treatment with activin A decreased the expression of epithelial marker E-cadherin and induced the mesenchymal markers N-cadherin and vimentin in HaCat cells. (B) Follistatin treatment promoted the expression of E-cadherin and blocked the expression of N-cadherin and vimentin in SCC-9 ZsGreen LN-1 cells. (C) Abrogation of activin A induced the expression of E-cadherin, whereas downregulated the production of N-cadherin and vimentin. (D) Representative images of aggregates formed by shControl and shINHBA cells in hanging drop assay. Silencing of activin A induced cell-cell adhesion, as revealed by larger aggregates in shINHBA cultures compared to those in shControl cultures. *p<0.05, **p<0.01, ***p<0.001, ****p<0.0001.

In the process of invasion and metastasis, tumor cells loss epithelial features and evoke mesenchymal phenotypes characterizing the EMT process. TGF-β and its family members are recognized promoters of EMT [12]. To gain insights into the regulation of EMT and invasion by activin A, we examined several features such as expression of epithelial marker E-cadherin and mesenchymal markers N-cadherin and vimentin, adhesive capacity, presence of filopodia and lamellipodia, motility and invasiveness. Reduced levels of E-cadherin mRNA were observed in HaCat cells treated with 10 and 100 ng/ml of activin A (Fig 7A). Concomitantly, activin A increased the expressions of N-cadherin and vimentin mRNA at concentrations 100 ng/ml (Fig 7A). Conversely, follistatin promoted the expression of E-cadherin mRNA whereas decreased the expression of N-cadherin and vimentin in SCC-9 ZsGreen LN-1 cells (Fig 7B). As revealed in Fig 7C, shINHBA target cells showed lower levels of N-cadherin and vimentin mRNA, and higher E-cadherin mRNA levels compared to shControl cells. The levels of those EMT markers were also analyzed at protein levels by western blot; similar changes were observed at the protein level (S3 Fig). In accordance with those results, our observations using phase contrast microscopy suggested that shINHBA cells have more cell-cell attachment. In order to confirm such observation, hanging drop assay was performed. Activin A knock down cells formed larger aggregates compared to control cells, indicating that activin A controls of cell-cell contacts via regulation of E-cadherin (Fig 7D).

**Fig 8:**
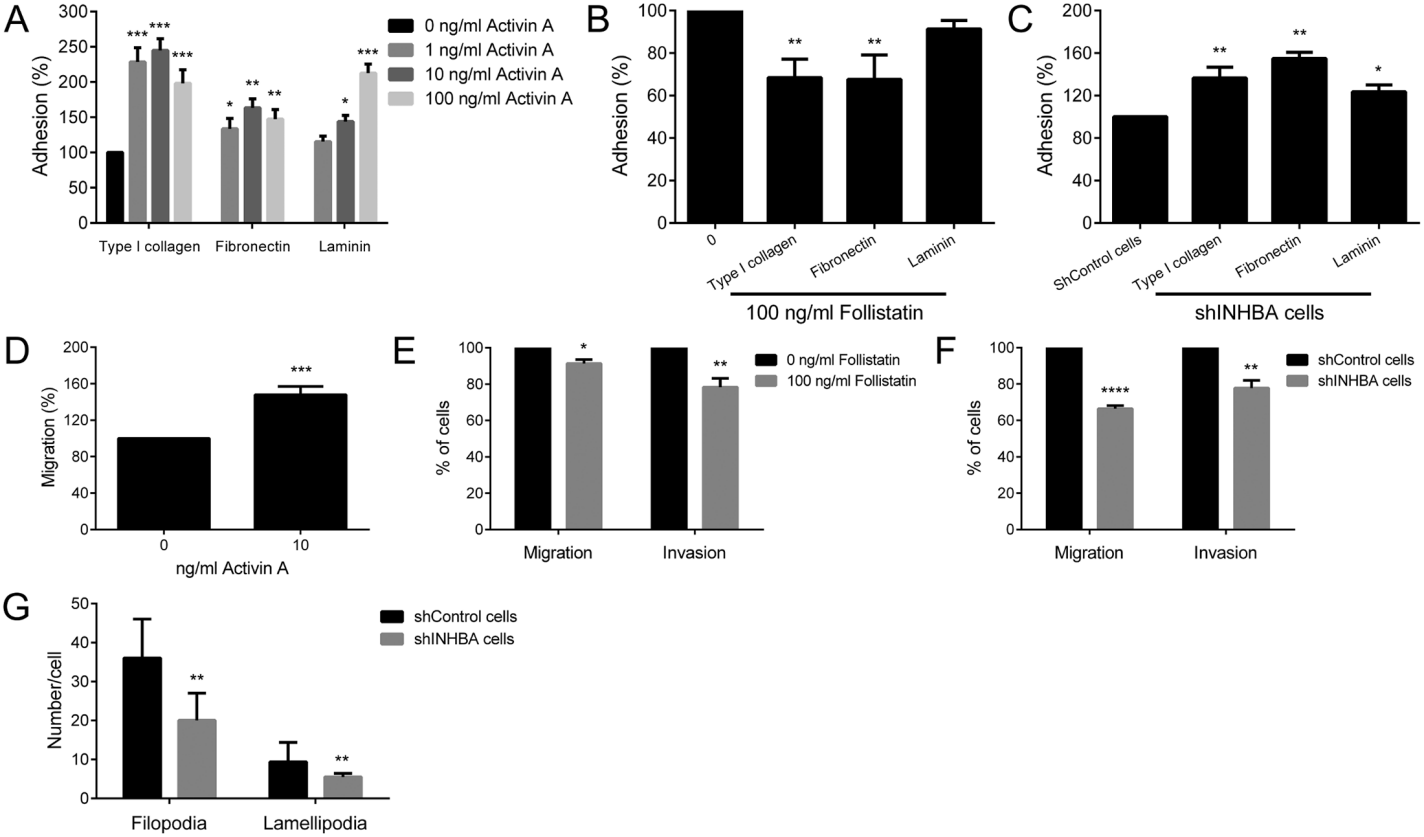
Activin A modulates the adhesion, migration and invasion of OSCC cells. (A) Activin A treatment induced significantly the adhesion of HaCat cells on surfaces coated with type I collagen, fibronectin or laminin. (B) Follistatin decreased significantly the adhesion of SCC-9 ZsGreen LN-1 cells o surfaces coated with type I collagen and fibronectin. (C) Activin A knockdown augmented the adhesion to coated-surfaces, as revealed by significantly higher adhesion of shINHBA cells compared with shControl cells. (D) Activin A induced significantly the migration of HaCat cells, whereas follistatin blocked it and also reduced significantly the invasion of SCC-9 ZsGreen LN-1 cells through Matrigel-covered surfaces (E). (F) The migration and invasion of shINHBA cells were significantly lower in comparison with shControl cells. (G) Knock down of activin A interferes with cytoskeleton organization, reducing filopodia and lamellipodia formation. The number of filopodia and lamellipodia was significantly lower in shINHBA cells than in shControl cells. *p<0.05, **p<0.01, ***p<0.001, ****p<0.0001.

Next, the effects of activin A on adhesive properties of the cells were investigated on surfaces coated with type I collagen, fibronectin and laminin. In general, activin A significantly increased the adhesion of HaCat cells on surfaces coated with type I collagen, fibronectin and laminin (Fig 8A), whereas follistatin reduced adhesion significantly to surfaces coated with type I and fibronectin (Fig 8B). Adhesion of shINHBA cells was significantly higher than shControl cells in all 3 coated-surfaces (Fig 8C). Activin A significantly augmented migration of HaCat cells (p<0.01, Fig 8D), whereas follistatin significantly inhibited the migration and invasiveness of SCC-9 ZsGreen LN-1 parental cells (p<0.05, Fig 8E). shINHBA cells showed significantly lower migration (p<0.01) and invasion (p<0.05) compared with control cells (Fig 8F). The number of filopodia and lamellipodia in shINHBA cells was also significantly lower compared with shControl cells (p<0.001, Fig 8G and S4 Fig).

### Downregulation of miR143/miR145 cluster is associated with INHBA overexpression in OSCC

**Fig 9:**
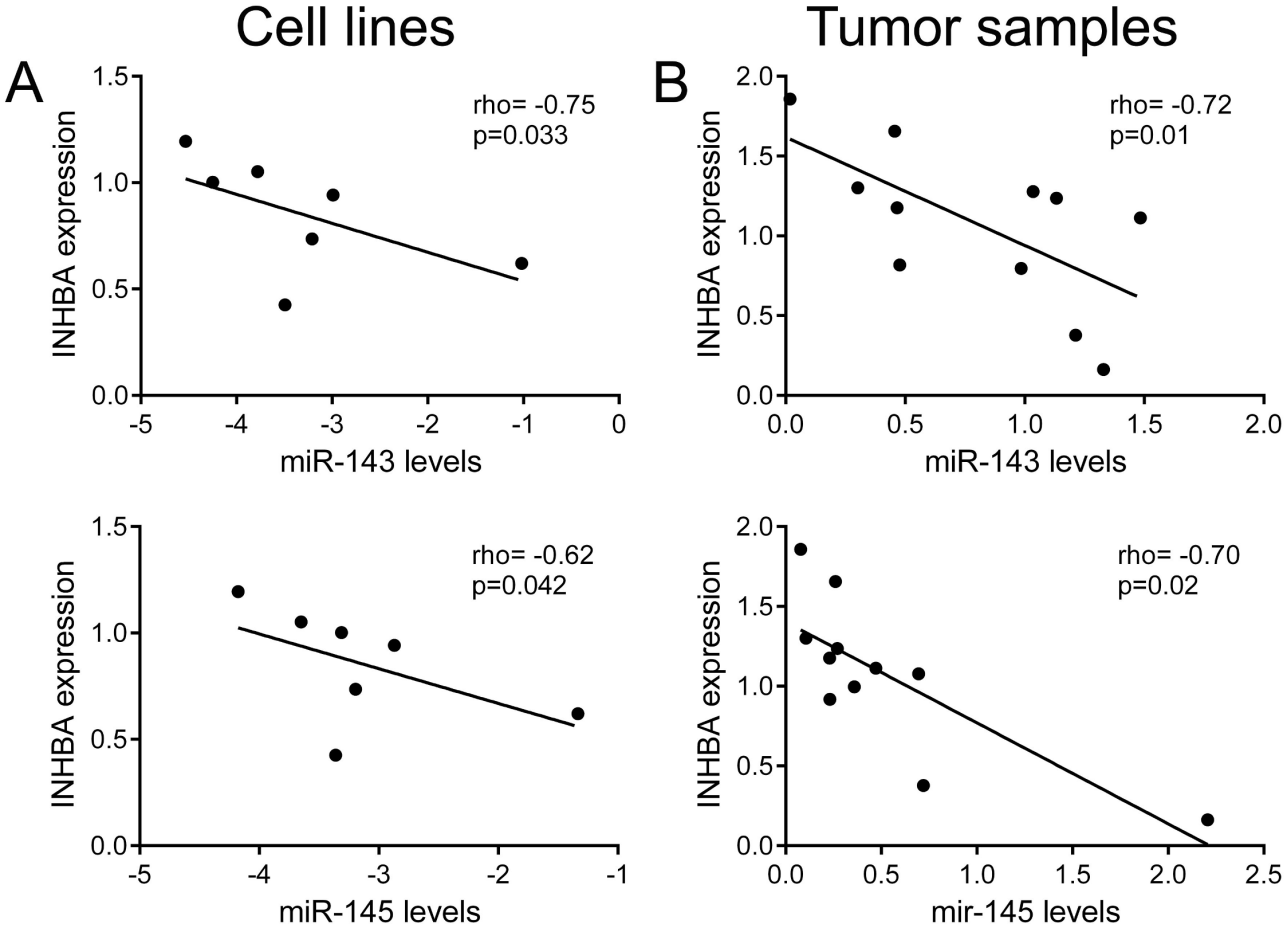
Expression of INHBA is inversely correlated with miR-143 and miR-145 expression in OSCC cells lines and tumor samples. (A) Spearman correlation analysis between the expressions of INHBA and miR-143 and miR-145 in 7 OSCC cell lines (A) and in 11 OSCC fresh samples (B). A significant inverse correlation was observed between both miR-143 and miR-145 and INHBA expression levels.

Since previous study showed that miR-145 regulates features similar to those regulated by activin A in OSCCs [13], and to gain insight into the molecular mechanism by which activin A is overexpressed in OSCCs, we determined the relationship between INHBA mRNA levels and expression of miR-143 and miR-145 in a series of OSCC cell lines and fresh tumor samples. For both microRNAs, a significant and inverse correlation with INHBA levels was observed in the cell lines (rho = -0.75 and p = 0.033 for miR-143, rho = -0.62 and p = 0.042 for miR-145, Fig 9A) and fresh tumor samples (rho = -0.72 and p = 0.01 for miR-143, rho = -0.70 and p = 0.02 for miR-145, Fig 9B).

**Fig 10:**
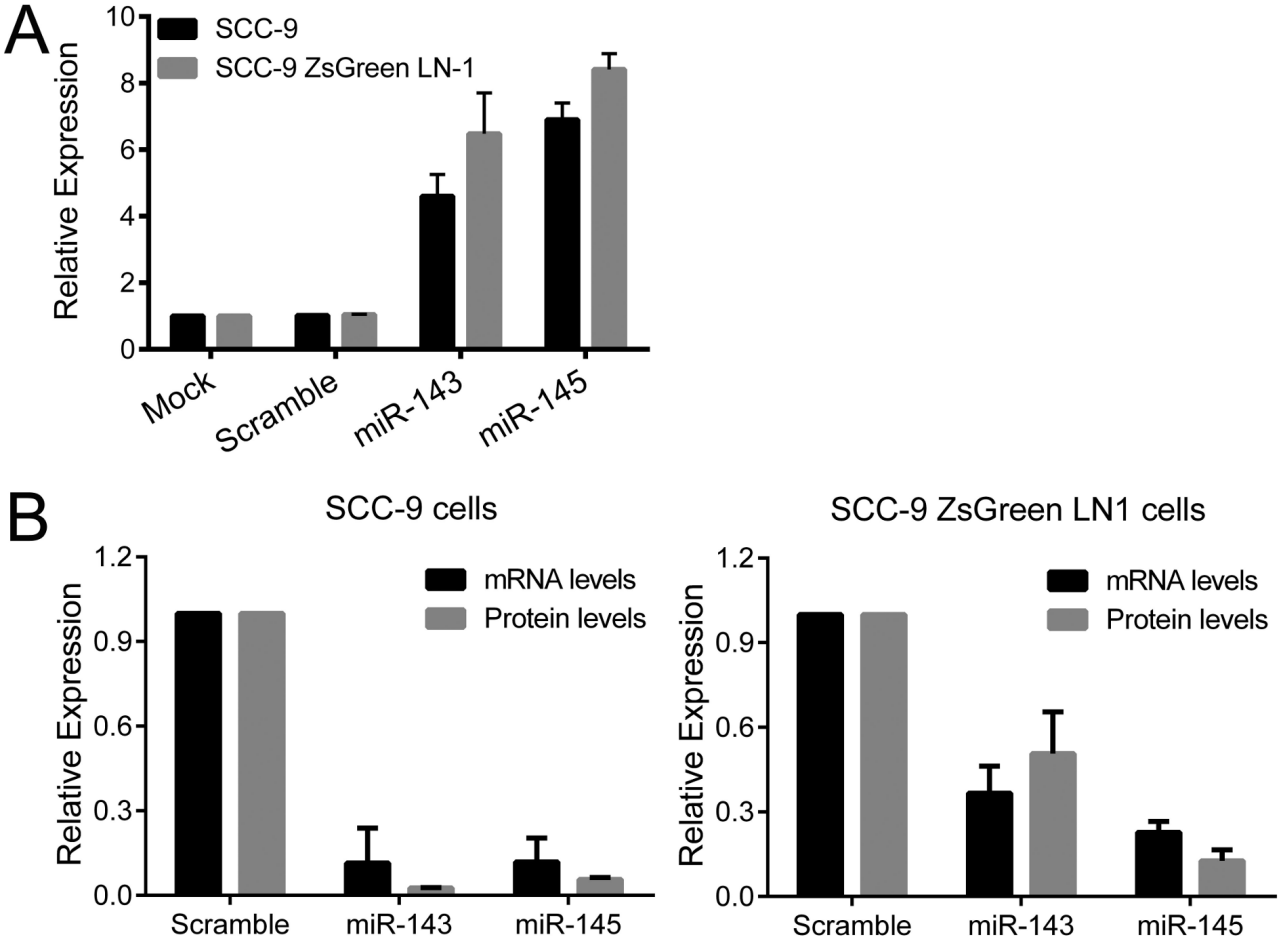
INHBA mRNA is target by miR-143 and miR-145. (A) SCC-9 and SCC-9 ZsGreen LN-1 cells were exposed to Scramble control or miR-143 and miR-145 mimics. qPCR analysis demonstrated that levels of miR-143 and miR-145 were dramatically increased after introduction of the mimics. (B) Levels of activin A mRNA and protein were clearly decreased in both OSCC cell lines, demonstrating that miR-143/miR-145 cluster regulates INHBA mRNA levels.

To determine whether miR-143 and miR-145 regulate INHBA mRNA, SCC-9 and SCC-9 ZsGreen LN-1 cells were transfected with miR-143 and miR-145 mimics. Fig 10A shows the efficiency of miR-143 and miR-145 transfection in SCC-9 and SCC-9 ZsGreen LN-1 cells, which reached 5 to 8-fold increased levels. The increase in miR-143 and miR-145 levels resulted in a concomitant decrease in activin A mRNA and protein, demonstrating that miR-143/miR-145 cluster regulates INHBA mRNA levels (Fig 10B).

## Discussion

Like other members of the TGF-β family, dual functions in cancer, both pro- and anti-tumorigenesis, have been attributed to activin A depending of the tumor development stage and cancer type [14–16]. Furthermore, whilst some studies have demonstrated a stimulation of apoptosis after activin A treatment [17–18], others showed that activin A can restrain apoptosis via downregulation of caspase 3 expression [19]. Activin A inhibited cell growth of breast cancer cells [20–21] and prostate tumors [22], but it promoted the proliferation of ovarian cancer cells [23–24]. Most studies, however, have shown that activin A is involved in the progression of numerous types of human cancers via its oncogenic roles. Activin A promoted migration and invasiveness of different cancer cells [25–28], which were concomitant with a down-regulation of E-cadherin, a marker of EMT [25–29]. Oral carcinoma cells in culture expressed activin A and knock down with specific siRNA against INHBA reduced growth and induced invasion of tumor cells [8]. It was also associated with poor prognosis in OSCCs [8]. Our interest in activin A is based on our recent studies demonstrating that OSCC cell proliferation is increased in response to high levels of activin A released by CAFs present in the tumor microenvironment [7], and that expression of activin A by OSCC cells can be useful for prognostication of OSCC of the tongue, revealing patients with occult lymph node metastasis and lower overall survival [6]. In the current study we further demonstrate that activin A is overexpressed in OSCCs in comparison with normal oral mucosa, and its expression is correlated with lymph node metastasis (N+), poor histopathological grade, and patients whose tumors overexpressed activin A had a worse prognosis, as revealed by shortened overall survival. The present study also revealed that activin A regulates essential phenotypes associated with malignant transformation, such as apoptosis, proliferation, adhesion, migration, invasion and EMT process. Furthermore, our data demonstrate that a possible mechanism of activin A mRNA overexpression in OSCC is downregulation of miR-143/miR-145 cluster. Together, these data support a causal role for activin A in OSCCs, and suggest that activin A expression might be helpful as a prognostic marker for patients with this disease.

Activin A is known to control proliferation and apoptosis in a number of contexts both in normal development and tumorigenesis [2]. Since during the expansion and differentiation of stem cell population activin A increases proliferation and decreases apoptosis [30], it is expected that deregulation of activin A in adult cells would have dire consequences, such as cancer development and progression. In prostate cancer, activin A plays a paradoxical role, because in low-grade tumors, apoptosis is increased by activin A, but in high-grade metastatic tumors, cells are no longer sensitive to activin A-mediated apoptosis [31]. A significant tolerance to Fas-induced apoptosis was also observed in esophageal carcinoma cells overexpressing activin A [3]. Similarly, the results presented in this study showed that high levels of activin A suppress apoptosis of OSCC cells, and the treatment of low-activin A expressing cells with activin A partially inhibited staurosporine-mediate apoptosis. In different cell lines activin A induces proliferation, which is antagonized by follistatin [8,23,24]. However, the mechanisms involved in activin A-promote proliferation of OSCC cells are not characterized. Our results showed that knock down of activin A results in reduction of proliferation and retention of the cells at G0–G1 phases of cell cycle, which were accompanied by increased levels of p16, p21 and p27, indicating the mechanisms involved in activin A cell growth control.

Inappropriate expression of activin A has been shown to modulate migration and invasion in different cell lines. Kang et al. [26] demonstrated that activin A promotes migration of prostate cancer cells through SMAD pathway and androgen receptor activation, thereby promoting bone metastasis. In an esophageal organotypic culture model, activin A treatment resulted in MMP-dependent invasion, requiring the presence of fibroblasts [28]. Furthermore activin A induced trophoblast cell invasion in parallel with the expression of N-cadherin, SNAIL and SLUG, however siRNA-mediated depletion of SNAIL or SLUG did not affect activin-induced N-cadherin expression [32]. Those data in conjunction with recognized functions of TGF-β family members in the regulation of EMT suggest that activin A may induce migration and invasion as an end point of EMT process. EMT is the biological process by which epithelial cells loss the cell-to-cell adhesions, gain the expression of mesenchymal proteins and enhanced migratory and invasive capacity, allowing tumor cells to acquire metastatic properties [23]. Although EMT is dependent of a series of cellular events, the reduction of E-cadherin expression in consonance with upregulation of mesenchymal proteins such as N-cadherin and vimentin have been the most common markers of EMT [33]. In cells depleted of activin A, we observed an induction of E-cadherin and a repression of N-cadherin and vimentin, in consonance with other EMT-relates features including alterations in the adhesive proprieties to ECM proteins, diminished cell-cell adhesion, reduced filopodia and lamellipodia formations and decreased migration and invasion. In support of these in vitro findings, high immunohistochemical expression of activin A was significantly correlated with lymph node metastasis. Therefore, it is possible that overexpression of activin A evokes the EMT process, an important phenotype for invasion and metastasis of malignant cells.

Although overexpression of activin A has been reported in several cancers, the mechanisms responsible for this overexpression are unknown. Our findings revealed that miR-143 and miR-145 are downregulated in OSCC cell lines and specimens, and their expressions are inversely correlated with INHBA levels. Additionally, we found that the transfection of miR-143 and miR-145 mimics in OSCC cells result in a downregulation of INHBA, suggesting that activin A overexpression in OSCCs is, at least in part, regulated by those microRNAs. Increasing evidences indicate that miR-145 and, more recently, miR-143 act a tumor suppressor cluster in numerous human cancers [34–36]. For example, ectopic expression of miR-143 significantly inhibited cell proliferation of non-small cell lung cancer cells [37] and migration and invasion in prostate cancer [38]. The levels of miR-145 decreased gradually, from normal to cancer, during breast and prostate cancer progression [39–40]. In addition, a previous study by Drebber et al. [41] showed that miR-145 downregulation may be an important molecular biomarker for early diagnosis of colon cancer, and its elevation in tumor tissues is predictive of good chemoradiotherapy treatment response. Recently, Shao et al. [13] reported that the upregulation of miR-145 resulted in a suppression of oral tumor cell proliferation, migration and invasion. Interestingly, in silico analysis indicates there are no putative conserved binding sites for either miR-143 or miR-145 in the 3’UTR of the INHBA transcript (data not shown), suggesting the repression observed here may be subsequent to the down-regulation of a direct target able to regulate activin A expression. Given the importance of miR-143 and miR-145 in cancer, particularly associated with activin A regulation, further studies shall be conducted to address their roles in oral carcinogenesis.

In summary, activin A shows pleiotropic functions affecting many cellular processes including proliferation, survival and invasion [2]. It has been postulated that deregulation of activin A leads to an out of context activation of those cellular functions, contributing to tumor initiation and progression. In the present study we show that overexpression of activin A in OSCCs, which is at least in part caused by downregulation of miR-143/miR-145 cluster, is correlated with lymph node metastasis and overall survival. Moreover, strong evidences revealed that activin A overexpression in OSCC cells enhances survival and promotes proliferation, via regulation of CDK inhibitors p16, p21 and p27, and migration and invasion, via promotion of EMT. Overall, our data suggest that deregulated expression of activin A in oral cells actively promotes tumorigenesis by control important phenotypes related to malignant transformation.

## Supporting Information

S1 Fig Expression levels of activin A in normal and malignant keratinocytes. Total RNA from cell lines were converted in cDNA and subjected to qPCR. INHBA mRNA levels were significantly higher in OSCC cell lines compared to the normal human epithelial cell line (HaCat), with exception of SCC-4.
(JPG)

S2 Fig Efficiency of activin A knockdown in SCC-9 ZsGreen LN-1 cells. Cells were transduced with lentivirus expressing shRNA sequences against INHBA (shINHBA cells) and control (shControl cells) as outlined in the methods. shINHBA cells showed a marked reduction in both mRNA and protein levels when compared with shControl cells.
(JPG)

S3 Fig Effect of activin A and follistatin on markers of epithelial-mesenchymal transition. Cells were treated with 100 ng/ml activin A (A) or follistatin (B) followed by western blot analysis for E-cadherin, N-cadherin and vimentin. While activin A induced epithelial-mesenchymal transition, follistatin blocked it as revealed by high amounts of E-cadherin and low of N-cadherin and vimentin.
(JPG)

S4 Fig Detection of filopodia and lamellipodia in shControl and shINHBA cells. Cells were labeled with Alexa Fluor 488 phalloidin and DRAQ5 to characterization of actin filaments and nuclei, respectively. Filopodia (arrowheads) and lamellipodia (arrow) were more abundant in shControl cells than in shINHBA cells.
(JPG)

S1 Table (DOCX)

S2 Table (DOCX)
