# Supplementary figures and images for: Low miR-143/miR-145 Cluster Levels Induce Activin A Overexpression in Oral Squamous Cell Carcinomas, Which Contributes to Poor Prognosis
Source: PLoS One. 2015 Aug 28;10(8):e0136599. doi: 10.1371/journal.pone.0136599 (PMC4552554; doi:10.1371/journal.pone.0136599)

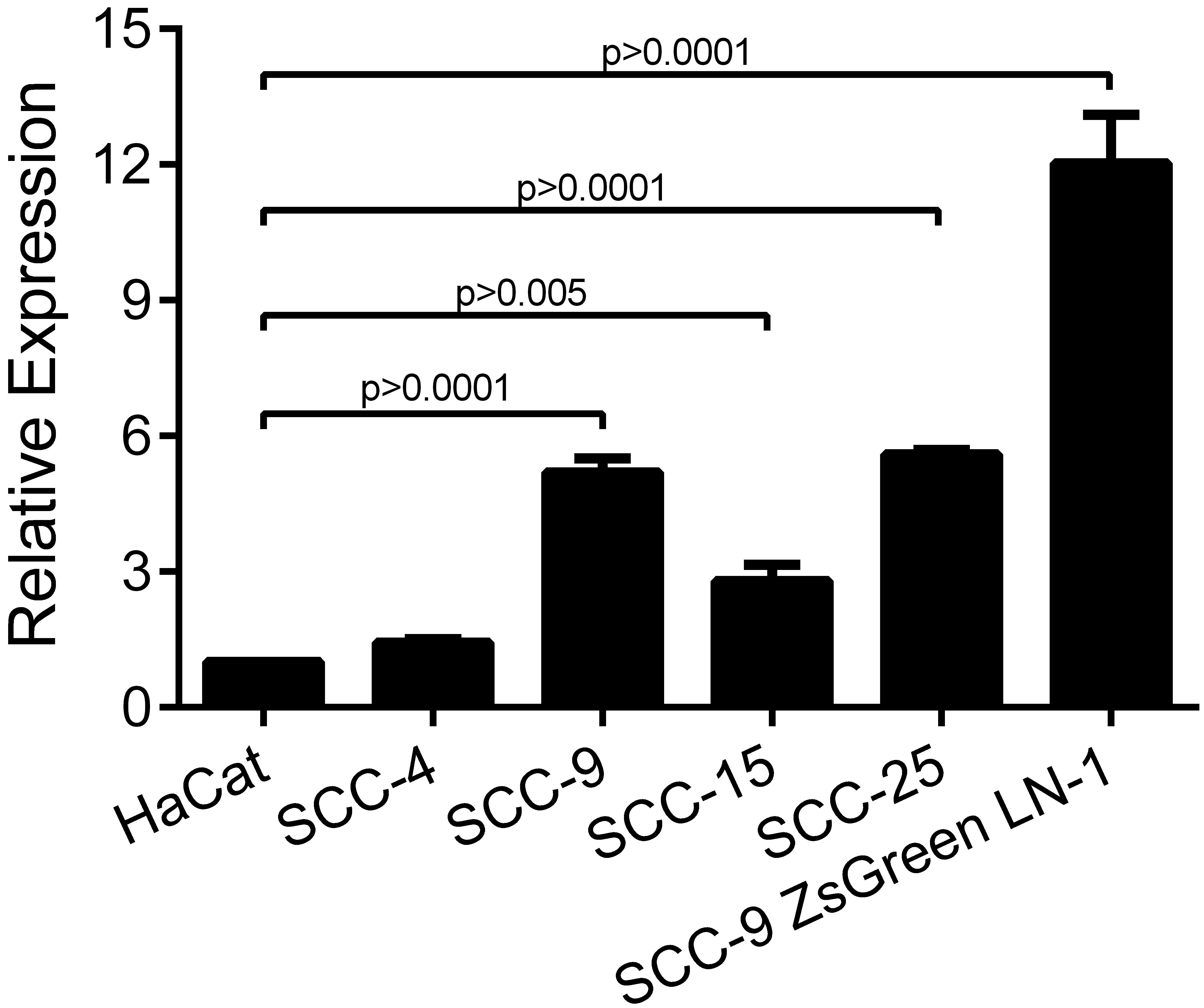

Supplement: S1 Fig — Total RNA from cell lines were converted in cDNA and subjected to qPCR. INHBA mRNA levels were significantly higher in OSCC cell lines compared to the normal human epithelial cell line (HaCat), with exception of SCC-4. (JPG) [file pone.0136599.s001.jpg]

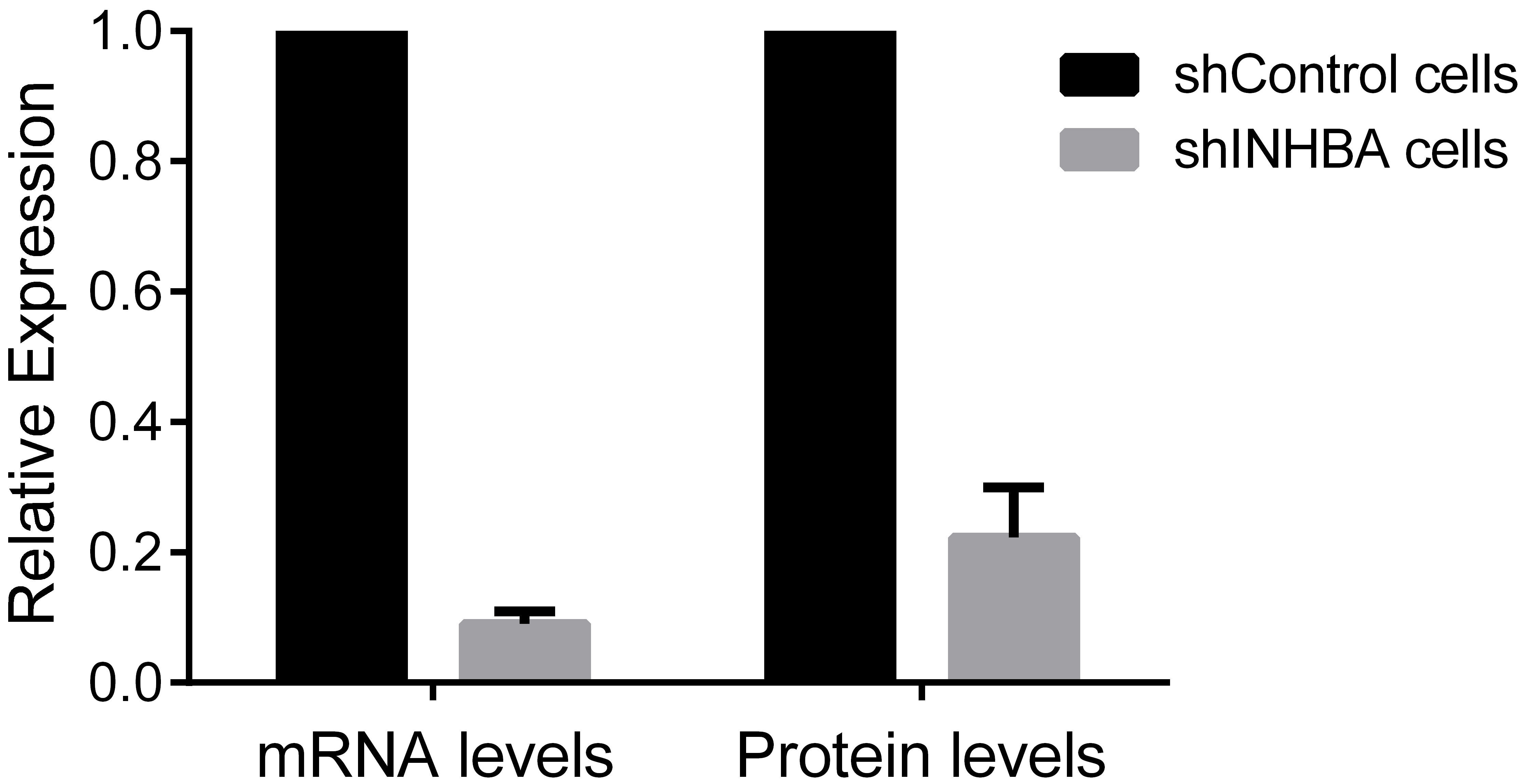

Supplement: S2 Fig — Cells were transduced with lentivirus expressing shRNA sequences against INHBA (shINHBA cells) and control (shControl cells) as outlined in the methods. shINHBA cells showed a marked reduction in both mRNA and protein levels when compared with shControl cells. (JPG) [file pone.0136599.s002.jpg]

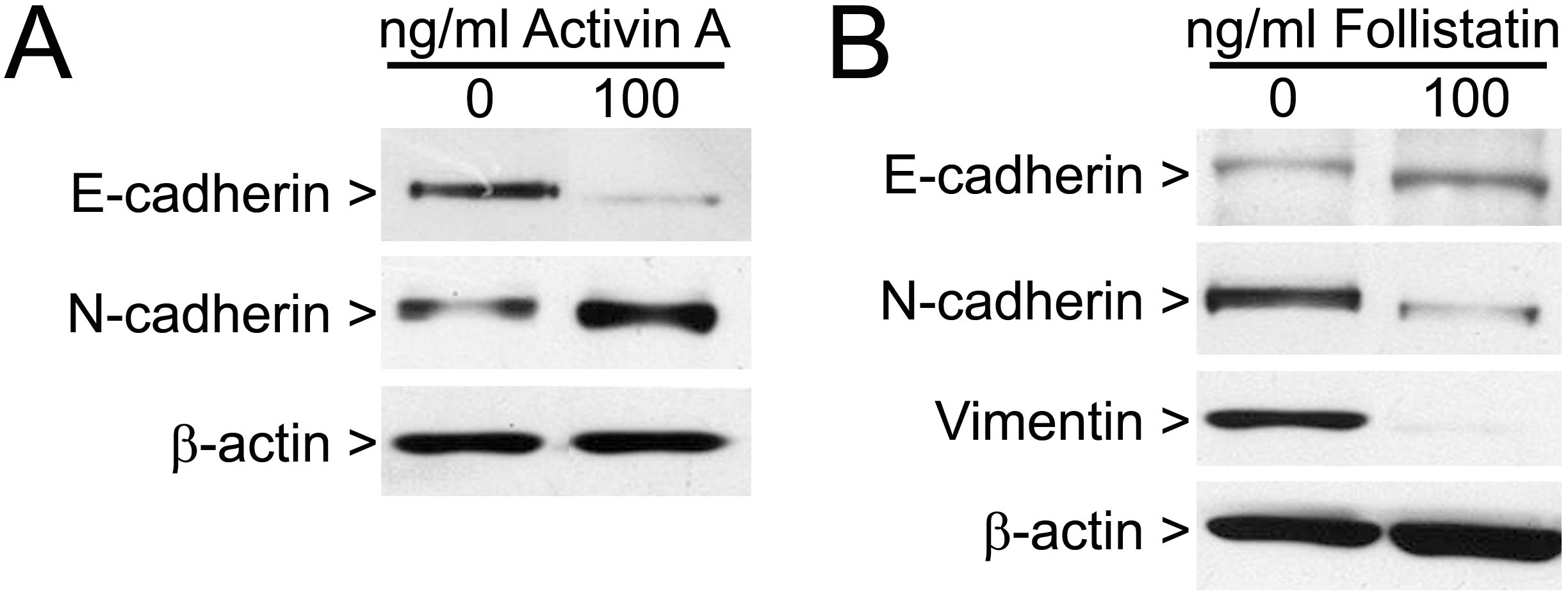

Supplement: S3 Fig — Cells were treated with 100 ng/ml activin A (A) or follistatin (B) followed by western blot analysis for E-cadherin, N-cadherin and vimentin. While activin A induced epithelial-mesenchymal transition, follistatin blocked it as revealed by high amounts of E-cadherin and low of N-cadherin and vimentin. (JPG) [file pone.0136599.s003.jpg]

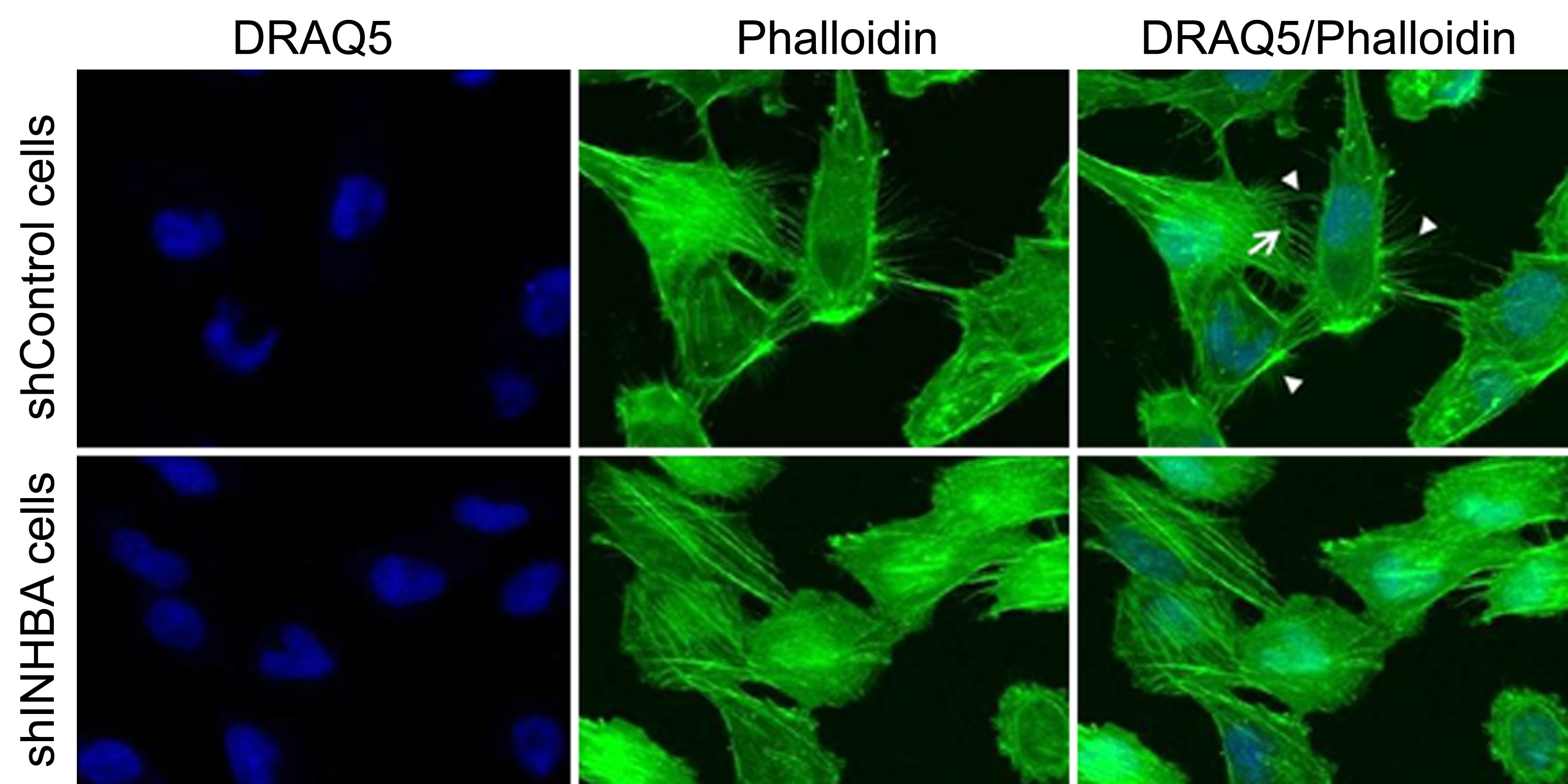

Supplement: S4 Fig — Cells were labeled with Alexa Fluor 488 phalloidin and DRAQ5 to characterization of actin filaments and nuclei, respectively. Filopodia (arrowheads) and lamellipodia (arrow) were more abundant in shControl cells than in shINHBA cells. (JPG) [file pone.0136599.s004.jpg]
