# Supplementary material for: Low miR-143/miR-145 Cluster Levels Induce Activin A Overexpression in Oral Squamous Cell Carcinomas, Which Contributes to Poor Prognosis
Source: PLoS One. 2015 Aug 28;10(8):e0136599. doi: 10.1371/journal.pone.0136599 (PMC4552554; doi:10.1371/journal.pone.0136599)
Supplement: S1 Table — (DOCX) [file pone.0136599.s005.docx]

S1 Table. Primers used in qPCR assays.

| Target | Forward (5’→3’) | Reverse (5’→3’) |
| --- | --- | --- |
| INHBA | CCCCTTTGCCAACCTCAAA | CATGGACATGGGTCTCAGCTT |
| E-cadherin | ACAGCCCCGCCTTATGATT | TCGGAACCGCTTCCTTCA |
| N-cadherin | TGGGAATCCGACGAATGG | CGTACGGCGCTGGGTATC |
| Vimentin | GGCTCGTCACCTTCGTGAAT | TCAATGTCAAGGGCCATCTTAA |
| PPIA | GCTTTGGGTCCAGGAATGG | GTTGTCCACAGTCAGCAATGGT |
