# Supplementary material for: Low miR-143/miR-145 Cluster Levels Induce Activin A Overexpression in Oral Squamous Cell Carcinomas, Which Contributes to Poor Prognosis
Source: PLoS One. 2015 Aug 28;10(8):e0136599. doi: 10.1371/journal.pone.0136599 (PMC4552554; doi:10.1371/journal.pone.0136599)
Supplement: S2 Table — (DOCX) [file pone.0136599.s006.docx]

S2 Table. Antibodies against cell cycle proteins and epithelial-mesenchymal transition markers used in the western blot reactions.

| Antibody | Clone | Company | Dilution |
| --- | --- | --- | --- |
| p16 | G-6 | Santa Cruz | 1:100 |
| p21 | C-19 | Santa Cruz | 1:200 |
| p27 | 57 | BD Biosciences | 1:500 |
| CDK2 | M2 | Santa Cruz | 1:15000 |
| CDK4 | C-22 | Santa Cruz | 1:25000 |
| CDK6 | C-21 | Santa Cruz | 1:1000 |
| Cyclin D1 | DCS-6 | Calbiochem | 1:200 |
| Cyclin E | M-20 | Santa Cruz | 1:1000 |
| Phospho-RB | Sc-16671 | Santa Cruz | 1:200 |
| E-Cadherin | Sc-7860 | Santa Cruz | 1.2000 |
| N-Cadherin | 8C11 | BD Biosciences | 1:2500 |
| Vimentin | 3B4 | Dako | 1:200 |
| β-actin | AC-15 | Sigma-Aldrich | 1:30000 |
